# Prevalence of the different Axis I clinical subtypes in a sample of patients with orofacial pain and temporomandibular disorders in the Andalusian Healthcare Service

**DOI:** 10.4317/medoral.20854

**Published:** 2015-11-30

**Authors:** Antonio Blanco-Hungría, Antonio Blanco-Aguilera, Elena Blanco-Aguilera, Rafael Serrano-del-Rosal, Lourdes Biedma-Velázquez, Alejandro Rodríguez-Torronteras, Rafael Segura-Saint-Gerons

**Affiliations:** 1 MD. Stomatologist. Andalusian Healthcare Service, Córdoba-Guadalquivir Healthcare District. Maimonides Institute for Biomedical Research IMBIC. University of Córdoba, Spain; 2 PhD in Odontology. MS in Orofacial Pain, Temporomandibular Disorders and Orthodontics. CEU San Pablo University; 3 Odontologist. MS in Oral Medicine, Surgery and Implantology. University of Santiago de Compostela, Spain; 4 PhD in Sociology. Research Scientist. Institute for Advanced Social Studies, Spanish National Research Council; 5 Sociologist. Senior Research Technician. Institute for Advanced Social Studies. Spanish National Research Council; 6 Epidemiologist. Andalusian Healthcare Service, Córdoba-Guadalquivir Healthcare District. University of Córdoba, Spain; 7 MD. Stomatologist. Andalusian Healthcare Service, Córdoba-Guadalquivir Healthcare District, Spain

## Abstract

**Background:**

The main objective of this paper is to analyze the prevalence of each of the different clinical subtypes of temporomandibular disorders (TMD) in a sample of patients with this pathology. In addition, a second objective was to analyze their distribution according to gender.

**Material and Methods:**

To this end, the results of 1603 patients who went to the Unit of Temporomandibular Disorders in the Córdoba Healthcare District because they suffered from this pathology were analyzed. In order to diagnose them, the Research Diagnostic Criteria for Temporomandibular Disorders (RDC/TMD) were applied, analyzing the different Axis I subtypes (myopathy, discopathy and arthropathy) and obtaining the combined Axis I for each patient and the relation of all these variables according to gender. The null-hypothesis test confirmed the lack of connection between the gender variable and the different subtypes in the clinical analysis, and between the former and the combined Axis I of the RDC/TMD.

**Results:**

The prevalence was high for the muscle disorders subtype in general, showing an 88.7% prevalence, while the presence of discopathies or arthropathies was much lower. Among discopathies, the most frequent ones were disc displacements with reduction, with 39.7% and 42.8% for the left and right temporomandibular joints (TMJ), respectively, while the prevalence of arthropathies was 26.3% for the right TMJ and 32.9% for the left TMJ. The bivariate analysis on the connection with gender reveals a *p*≥ 0.05 value for the muscle and arthralgia subtypes.

**Conclusions:**

The patients seen at the TMD Unit where mostly middle-aged women whose main clinical axis subtype was the muscle disorder subtype. For their part, both discopathies and arthropathies, although present, are much less prevalent.

**Key words:**RDCTMD, axis I, orofacial pain, temporomandibular disorders, gender.

## Introduction

The term temporomandibular disorders (TMD) defines a heterogeneous group of pathologies affecting the temporomandibular joint, the jaw muscles, or both (1). The main symptom of such disorders is localized pain in the orofacial region, which is defined by the International Association for the Study of Pain (IASP) as “an unpleasant sensory and emotional experience associated with actual or potential tissue damage, or described in terms of such damage”. In addition to pain, patients might also present other symptoms such as joint sounds (clicking and crepitus), which can, in turn, be related to alterations or limitations in mandibular dynamics.

Since when Costen described this symptoms in the last century, there have been certain authors who have proposed different systems for taking an anamnesis and examining the symptoms and signs caused by this pathology, with the aim of obtaining a diagnostic classification of the patients who suffer from it and to obtain specific clinical subtypes in order to be able to standardize future epidemiological studies.

Along these lines, Helkimo *et al*., carried out a series of epidemiological studies on patients with TMD, whose first conclusions were the high prevalence of signs and symptoms and their different distribution according to gender and age (2,3). However, as regards their etiology, he did not find any predominant factor, but he did find a certain correlation among the degree of TMD, the general health condition and the number of residual teeth. He also created an index of general temporomandibular dysfunction in order to shed more light on the dark aspects of the etiology and the course of the dysfunctional diseases of the masticatory system, applying a common methodology that makes it possible to analyze different populations (4).

Fricton *et al*., also developed another index, which they called Craniomandibular Index, which complied with the requirements of high sensitivity and specificity (5). To this effect, they analyzed a series of signs, including the presence of joint sounds, alterations in mandibular dynamics and pain when palpating the masticatory and craniocervical muscles. They concluded that the musculoskeletal disorders of the stomatognathic system were the cause of most diagnoses of chronic orofacial pain. The most common signs of these disorders are stiffness, limited or deviated range of motion or joint sounds (6).

At the end of the 20th century, the diagnostic classification proposed by Dworkin and Le Resche (7) prevailed, which, on the basis of a dual clinical and biopsychosocial axis, obtained, through a series of diagnostic algorithms, the mandibular limitation and the psychological aspects related to depression, anxiety and somatization typical of these patients (8).

The subsequent development of the criteria called Research Diagnostic Criteria for Temporomandibular Disorders (RDC/TMD), developed by a multicenter consortium gathering numerous specialists from different countries, has enabled a very wide range of epidemiological studies (9-12), on the basis of high reliability and sensitivity (13,14). In Spain, the Unit of Orofacial Pain and Temporomandibular Disorders of the Córdoba-Guadalquivir Healthcare District, which belongs to the Andalusian Healthcare Service, adopted these criteria in 2006 as a tool for anamnesis and exploration, in order to comprehensively assess and diagnose its patients, as well as as a valid tool to carry out epidemiological studies (15).

The objective of this study is to describe the different clinical subtypes, and their connection with gender, of a wide sample of patients with OP and TMD seen at the Andalusian Healthcare Service’s specialized unit for diagnosing and treating primary care patients with TMD and OP, to later compare them with other national and international units.

## Material and Methods

The population of the study is made up of 1622 patients who were referred to the Unit of Temporomandibular Disorders and Orofacial Pain of the Córdoba-Guadalquivir Healthcare District, which belongs to the Andalusian Healthcare Service. The study was carried out between January 2005 and October 2012, and all subjects of the population were referred by different healthcare specialists: family doctors, public healthcare odontologists and other specialist doctors, such as otolaryngologists, neurologists or maxillofacial surgeons.

All of them were applied the inclusion and exclusion criteria before taking their medical history and examining them, in accordance with the international standards included in the RDC/TMD’s Spanish version, validated in 2002 by González, and reviewed in the International RDC/TMD Consortium´s website; and its future directions developed by Anderson and Gonzalez (16). The criteria for inclusion were: being 18 or over and having reported some of the following signs or symptoms: pain in the TMJs or in the masticatory musculature; limited or restricted range of motion when opening or closing the mouth, or in lateral excursions; or joint sounds, with or without pain.

In turn, the exclusion criteria were: suffering from systemic rheumatic disease (with the exception of fibromyalgia and rheumatoid arthritis), or neurological or autoimmune diseases; patients who had undergone TMJ surgery or head and neck radiation treatment; pregnant patients; patients treated with narcotic analgesics, muscle relaxants, antidepressants, NSAIDs, or corticosteroids; and drug-dependent patients. The patients who did not wish to sign the compulsory informed consent form approved by the ethics committee of the Reina Sofía Teaching Hospital were also excluded. Thus, out of the 1622 TMD patients who were referred for diagnosis, 15 were excluded from the study because they were under 18, while 6 were excluded because they refused to sign the informed consent form or because they did not fill in the questionnaire, leaving a total of 1603 patients as the sample for this research.

Once the patients filled out the questionnaires, they were clinically examined in accordance with the RDC/TMD guidelines, with the aim of guaranteeing intra- and inter-examiner reproducibility (17). The clinical data of the RDC/TMD Axis I were obtained by a clinical researcher with expertise in examination, diagnosis and treatment of OP and TMD, with 30 years of experience and reliability in the diagnosis and treatment of TMD, who has taken part in numerous studies on this topic, as proposed by the scientific literature (18).

The three subgroups making up the RDC/TMD’s Axis I were obtained following the following parameters:

I Muscle disorder subgroup

Ia) Myofascial pain: Report of pain in the jaw, temples, face, preauricular area or inside the ear at rest or during function. Pain reported by the patient in response to palpation of at least 3 of the following muscle sites (right side and left side count as separate sites for each muscle): posterior temporalis, middle temporalis, anterior temporalis, origin of masseter, insertion of masseter, posterior mandibular region, submandibular region, lateral pterygoid area, and tendon of the temporalis. At least one of the painful sites must be on the same side as the complaint of pain.

Ib) Myofascial pain with limited opening: Myofascial pain as deﬁned in Ia, together with unassisted mandibular opening <40 mm, or assisted opening (passive stretch) ≤5 mm greater.

Ic) No pathology.

II Disc displacement subgroup

IIa) Disc displacement with reduction: Reciprocal clicking in TMJ (click on both vertical opening and closing that occurs at point ≥5 mm greater interincisal distance on opening than closing and is eliminated on protrusive opening), reproducible on 2 out of 3 consecutive trials.

IIb) Disc displacement without reduction with limited opening: History of signiﬁcant limitation in opening; maximum unassisted opening ≤35 mm; contralateral excursion <7 mm and/or uncorrected deviation to ipsilateral side on opening; absence of joint sound or presence of joint sounds not meeting criteria for disc displacement with reduction.

IIc) Disc displacement without reduction, without limited opening: History of signiﬁcant limitation of mandibular opening. Maximum unassisted opening >35 mm. Passive stretch increases opening by 5 mm over maximum unassisted opening. Contralateral excursion ≥7 mm. Presence of joint sounds not meeting criteria for disc displacement with reduction. In those studies allowing images, imaging conducted by either arthrography or magnetic resonance reveals disc displacement without reduction.

IId) No pathology.

III Arthralgia, osteoarthritis, osteoarthrosis subgroup

IIIa) Arthralgia: Pain in one or both joint sites (lateral pole and/or posterior attachment). One or more of the following self-reports of pain: pain in the region of the joints, pain in the joint during maximum unassisted opening, pain in the joint during assisted opening, and pain in the joint during lateral excursion. For a diagnosis of simple arthralgia, coarse crepitus must be absent.

IIIb) Osteoarthritis of the TMJ: Arthralgia as deﬁned in IIIa. Either coarse crepitus in the joint or radiologic signs of arthrosis.

IIIc) Osteoarthrosis of the TMJ: Absence of all signs of arthralgia. Either coarse crepitus in the joint or radiologic signs of arthrosis.

IIId) No pathology.

Once the subgroups have been defined, the RDC/TMD classification system allows many different diagnoses, with the possibility of classifying the patient as presenting no pathology; presenting only a muscle, disc or joint disorder; or presenting several subtypes at once (muscle and disc pathologies at the same time; muscle and joint pathologies; joint and disc pathologies; or all of them at once: muscle, disc and joint pathologies).

The statistical analysis of the different Axis I subtypes was obtained through frequencies and percentages, while the chi-squared test was performed in order to compare the gender prevalence among the different diagnoses. All the statistical procedures were carried out using r2 statistical software. All the participants signed an informed consent form and the study was approved by the ethics committee of the Reina Sofía Teaching Hospital.

## Results

Out of the 1603 patients, 1345 (83.9%) were women, with a mean age of 45.5 ± 15.8 years, while 258 were men, with a mean age of 44± 17.4, with an age range from 18 to 82. The female-to-male ratio is 4.5:1.

 Table 1 shows the different diagnostic subtypes in the clinical axis, with the different sample sizes for each of the three subgroups.

**Table 1:**
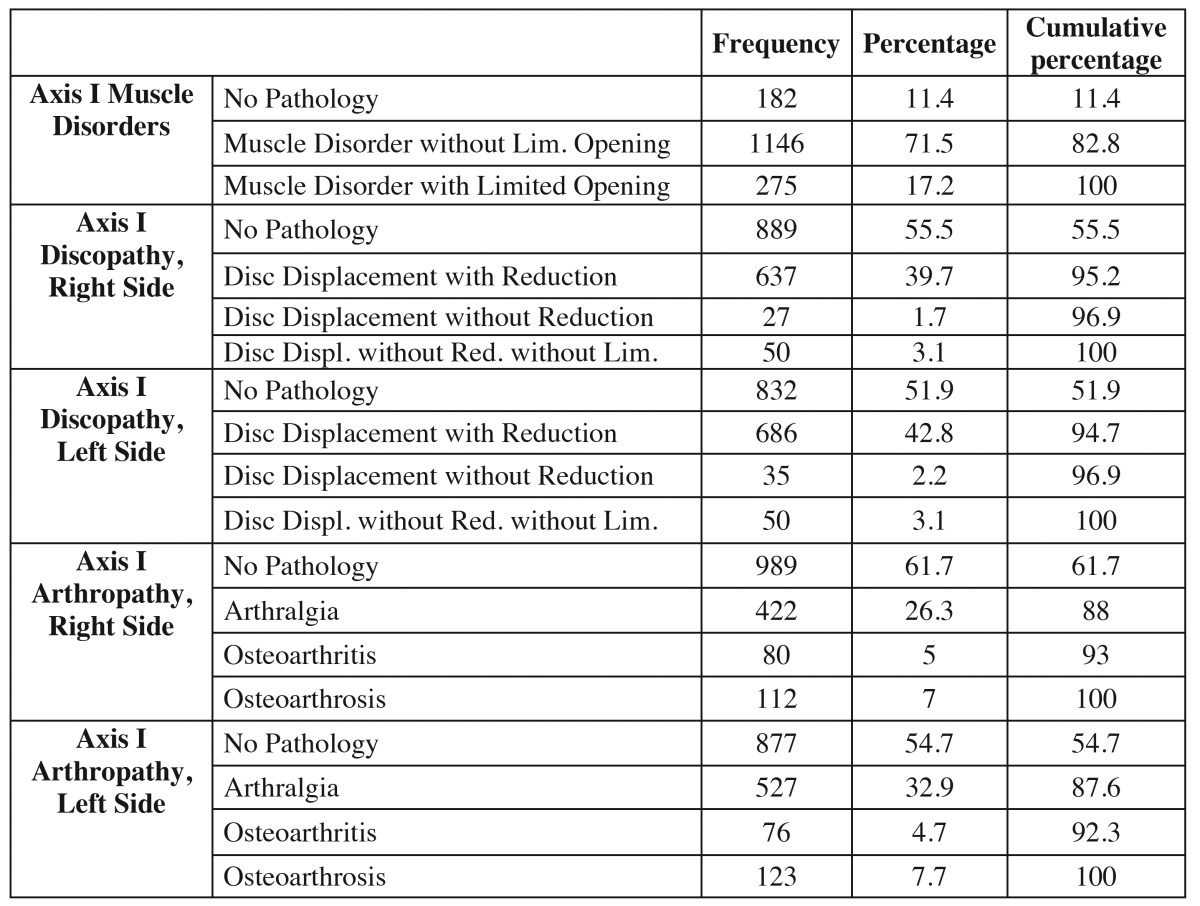
Distribution by frequency and percentages of all the RDC/TMD Axis I subgroups.

The distribution of the different Axis I subgroups by frequency and percentages reveals that the Axis I group I diagnosis (muscle disorders) was positive in 88.7% of the sample, with the subgroups distributed as follows: 1176 (71.5%) patients are included in the diagnostic group reporting muscle pain with no limited opening (Ia), while 275 (17.2%) patients report pain with limited opening (Ib). On the contrary, 11.35% of patients did not show the clinical algorithms to be defined as having muscle pain.

In the group II diagnosis (disc displacements), the signs and symptoms related to discopathies in the left TMJ were more prevalent (48.1%), while the right TMJ is 3% less prevalent (45.5%). Refining the analysis a little, if we focus on each of the subgroups we observe that the no pathology subgroup (IId) comprises 889 patients (55.5%) for the right TMJ and 832 patients (51.9%) for the left TMJ. The IIa subgroup (disc displacement with reduction) comprises 637 patients (39.7%) for the right TMJ and 686 (42.8%) for the left TMJ. The IIb subgroup is the least frequent, comprising 27 patients (31.7%) for the right TMJ and 35 (2.2%) for the left TMJ. The last subgroup of subtype II (IIc) shows a frequency of 50 patients (3.1%) for both joints.

The group III results reveal a similar pattern, where the most prevalent subgroup was that of patients with no pathology (IIId), with 989 (61.7%) and 877 (54.7%) cases for the right and left TMJs, respectively. The following group reveals half as many cases, which would be the arthralgia group, with 527 (32.9%) and 422 (26.3%) cases, but in this case the left TMJ is more prevalent. Just like in the second diagnostic group, the less frequent subgroup in intra-articular disorders is subgroup IIIb (osteoarthritis), with 80 (5%) patients for the right TMJ and 76 (4.7%) cases for the left TMJ. Lastly, the IIIc subgroup (osteoarthosis) shows 123 (7.7%) cases for the left TMJ and 112 (7%) for the right TMJ.

**Figure 1:**
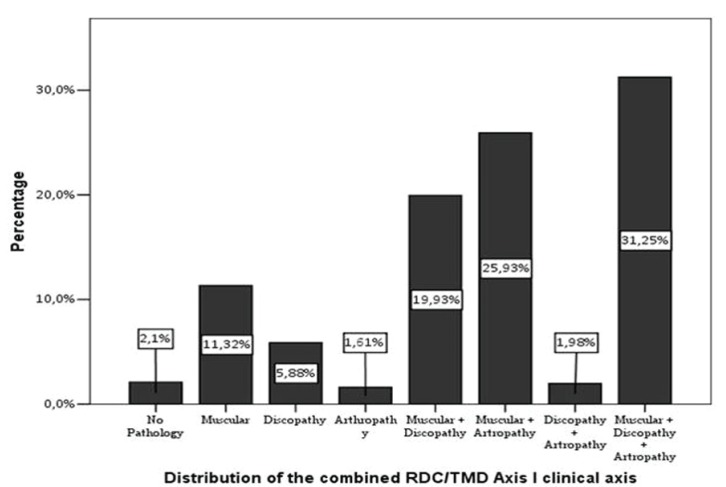
Distribution of the combined RDC/TMD Axis I clinical axis.

The frequency of the multiple diagnoses is shown in figure 1.

The group bringing together the three muscle groups and discopathy and arthropathy is the most prevalent, with 508 cases, 31.6% of the total of the sample, followed by the subgroups where the muscle disorder is connected to discopathies or arthropathies, with 321 cases (20%) and 411 (25.6%), respectively.

On the contrary, the less prevalent groups are pure arthropathies, with a frequency of 22 cases (1.37%), followed by the group comprising the association of discopathies and arthropathies, with 31 cases (1.9%). There is also a group with 26 cases (1.62%) of patients with no pathology.

 Table 2 shows the breakdown of the prevalence by gender for each subgroup and joint, confirming that the results, just like the ones included in  Table 1, show a high prevalence of muscle pathologies in both genders in the discopathy and arthropathy subgroups, with a lower prevalence than the no pathology group.

**Table 2:**
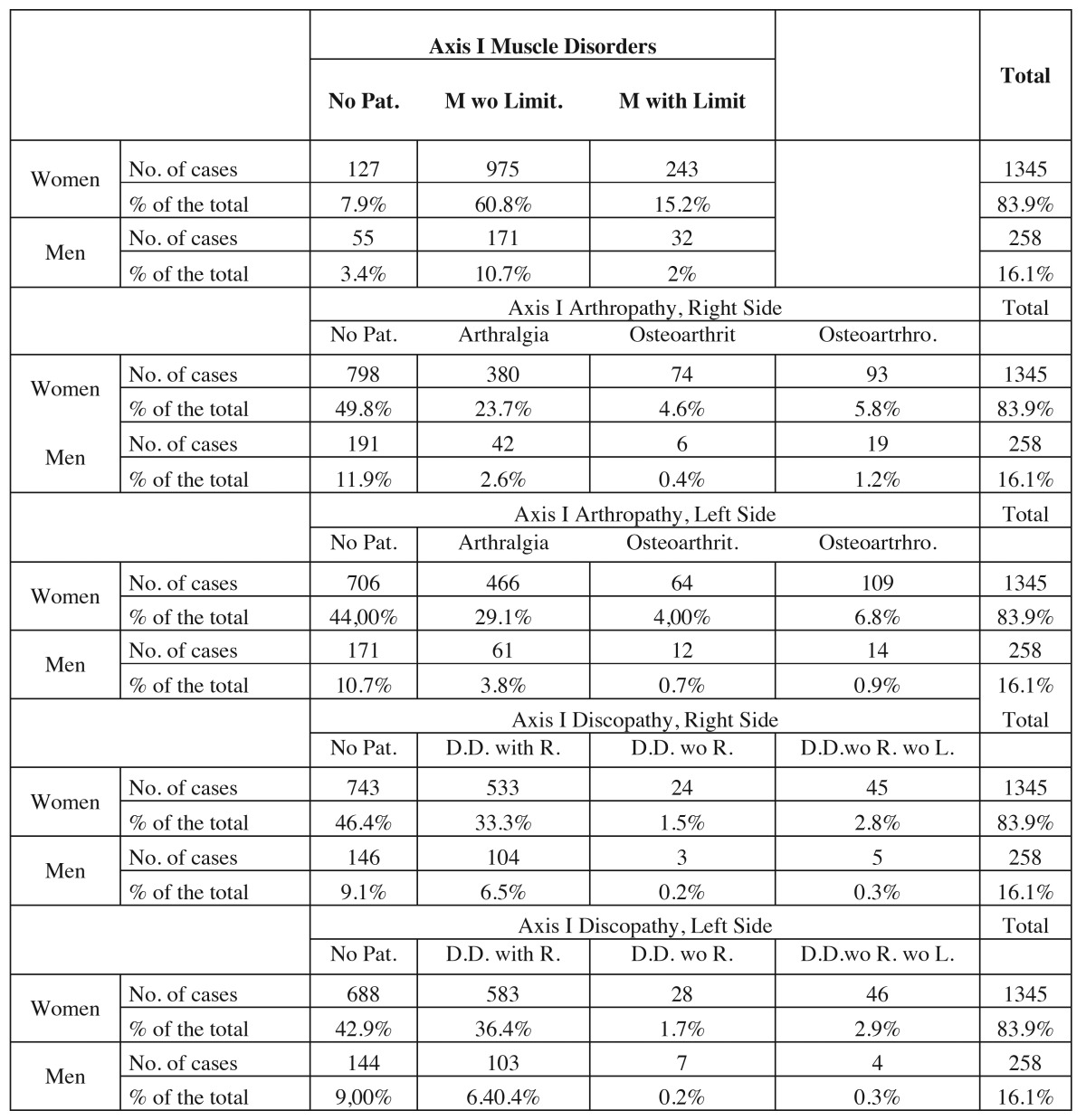
Distribution of the relation among the different subgroups according to the gender variable.

**Figure 2:**
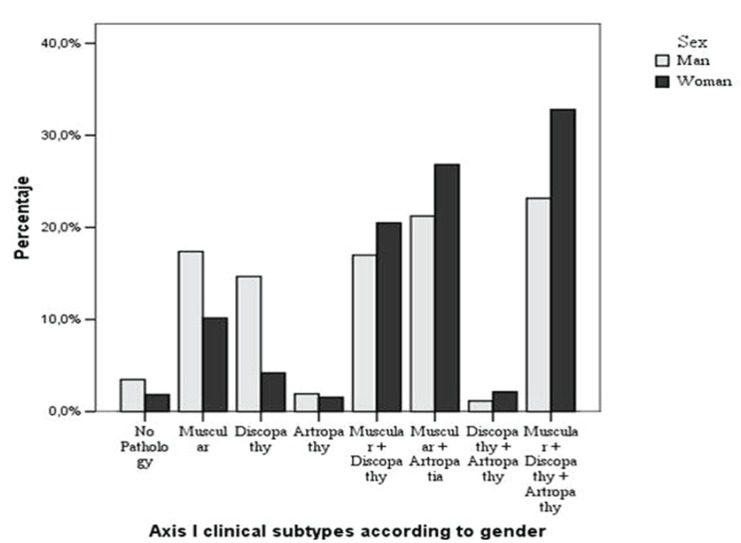
Distribution of the combined Axis I according to gender.

Figure 2 shows the distribution of the final combined Axis I, with the frequency of the seven Axis I groups for each patient, as well as of the no pathology group, which, as may be noted, like in the case of the patients with just one pathology, includes a higher percentage of men than women, while that percentage is inverted in the groups with different associated subgroups.

 Table 3 shows the results of a bivariate analysis between the different pathology groups analyzed in Axis I as far as the gender variable is concerned, revealing an association between that variable and the muscle and arthropathy subgroups of each patients’ right and left joints. On the contrary, the result of the chi-squared was not significant as regards the relation between gender and the variables related to disc displacements in each joint.

**Table 3:**
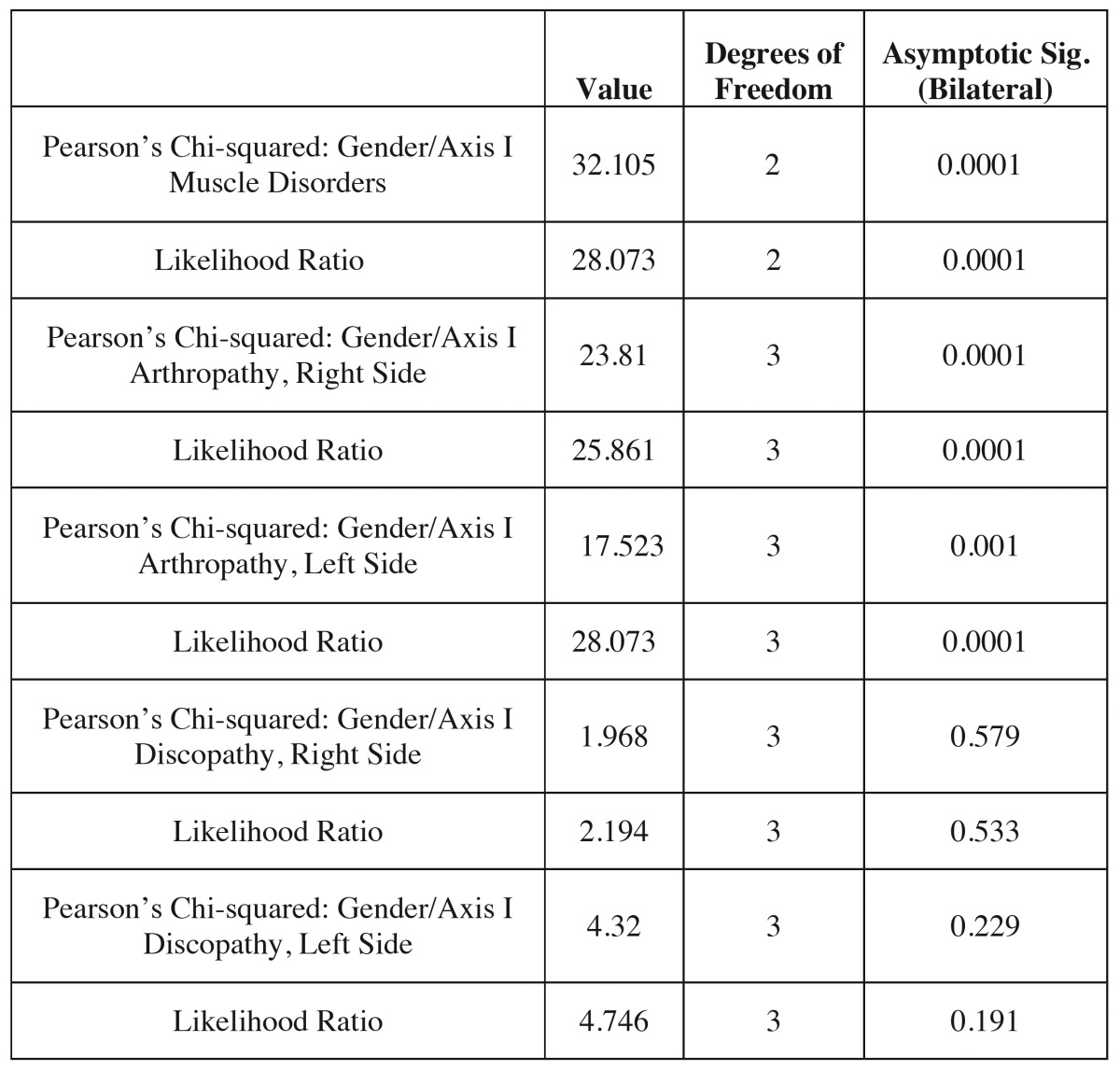
Qualitative analysis of the Axis I clinical subtypes according to gender.

## Discussion

The international standardization of the RDC/TMD criteria, facilitating the comparison of results among the different research units specialized in this matter, together with the fact that this protocol provides a clear distinction among the Axis I subgroups, has allowed us to study the different TMD pathologies separately. They differ to such an extent that, as some authors have suggested, they could have a different connection with the sociodemographic and/or etiological factors (19) traditionally associated with these disorders.

This is why the use of the RDC/TMD’s version 1 is recommended in all the methodological designs, also as an effective taxonomic classification to study this type of patients. However, there have been critical voices from the beginning, since this taxonomic classification had the deficiency of not including all the taxonomic subgroups of the TMD pathologies, according to the criteria of the American Academy of Orofacial Pain (AAOP). The Diagnostic Criteria for Temporomandibular Disorders does include them, but only the English language version has been validated (20).

The analysis of the literature reveals that this type of pathology is relevantly connected to the female gender, as the proportion is higher among women than among men, with a ratio ranging from 3.1:1 to 5:1. The ratio in our sample is closer to the latter, with a presence of 4.5 women for each man. In addition, the age variable in our sample, whose mean age is 45 years, is slightly over the average age in other studies, ranging from 30 to 40.

The detailed analysis of each of the subgroups reveals that the most prevalent group is muscle disorders, either with or without limited opening, with 71.5% and 17.1% respectively, as opposed to 11.4% who had no muscle pathology. The high prevalence is in line with previous studies that analyzed a sample of TMD patients, like List *et al*. (9), who compared two interculturally different groups of patients, which revealed that the group of Swedish patients showed a muscle disorder prevalence of 50% and 26% for subgroups Ia and Ib, as compared to 40% and 26% in the North American group. Also Barros, in a Brazilian population, stated that 50.6% and 26.5% (Ia and Ib) of the patients suffered from myofascial pain, with and without limited opening respectively, which coincides with the results of our muscle disorder group.

On the contrary, other authors like Manfredini (21) in Padua, in a sample of 520 patients, found that 56.4% of the sample had a muscle disorder (broken down into 36.5% of patients with pain without limited opening and 19.9% of patients with a muscle disorder with limited opening), which is far from the 88.6% found in our research. In another study carried out in Israel by Winocur (22), 65% of patients had myofascial pain with and without limited opening, a percentage higher than Manfredini’s, but 20% lower than ours.

In the subgroup of disc displacements with reduction, our results were 39.5% vs. 42.7% for the group of disc displacements with reduction in the right and left sides respectively, while the percentage for the groups of acute/chronic disc displacement without reduction was 4.8% vs. 5.3% for the right and left sides, respectively. If compared with the Italian population, they showed 42% of discopathies altogether, broken down into 30.4% for disc displacements with reduction and 11.6% for disc displacements without reduction, or, in other words, blocks. These results are similar to those obtained by Winocur in Israel, with 36.2% for DDWR and 20.9% for DDWOR.

On the contrary, in this subgroup other authors like John (23) report similar results to ours for DDWR, with 44.2%, slightly higher than our results, but they report results that double ours for DDWOR (11.1%). These data are similar to those obtained by Reismann (24) for the first subgroup, with 43.3% for DDWR, while they report 8.2% for DDWOR, which confirms that, just as is the case in our sample, the most prevalent group is the one with no disc pathology.

The high variability might be due not only to discrepancies when registering the data, but also to the fact that other sounds more associated with ligament laxity along with joint sounds (popping) may be interpreted as signs of joint sounds (clicking).

The last subgroup, referring to arthralgia (pain in joint sites with no associated sounds), was the most prevalent result among patients with a pathology, with 26.4% and 32.7% (right vs. left) respectively, followed by 5% and 4.8% for pain associated with crepitus (osteoarthritis), and finally 7% and 7.7% for presence of crepitus (osteoarthrosis). These results are different to those obtained by John (23), who reports 33.2%, 3.6% and 3.4% for each of the values of the Axis I third subgroup, while the rest of the authors report a lower prevalence of the arthropathy subgroups, which, altogether, do not reach 25% of patients.

In a review accompanied by a meta-analysis carried out by Manfredini *et al*. (12), where they analyzed 15 papers, the data reveal an average prevalence of 45.3% for the first group referring to muscle disorders, and 41.1% for the discopathy group altogether, while the prevalence for group III (arthropathies) is 30.1%. If we analyze the order of prevalence of all the subgroups, the most prevalent in the meta-analyses were Ia, with 34%; IIa, with 41.5%; and IIIa, with 34.2%.

In this review, Manfredini also analyzed 6 studies with samples of the general population and he reported mean values for each subgroup with a prevalence from 6% to 13.3% for muscle disorders, from 8.9 to 15.8% for discopathies, and 8.9% for arthropathies. Analyzing the order of prevalence of the subgroups, Ia obtained 9.7%, IIa obtained 11.4% and IIIa obtained 2.6%. This allows us to assess the great difference between the prevalence in the general population and the values obtained in the meta-analysis of samples of patients and our results, confirming the greater range of the latter.

Analyzing the percentage values of the combined distribution of the subgroups for each patient, we observe that the most prevalent groups are muscle disorder plus discopathy and arthropathy, followed by muscle disorder plus arthralgia, and muscle disorder plus disc displacement. On the contrary, the less prevalent are pure arthropathies and discopathies. These results do not coincide with those reported by Manfredini (21), where he showed that the most prevalent subgroup was muscle disorder plus discopathy (20.1%), closely followed by pure muscle disorder (19.9%). His results are clearly different to those of our sample, where the combination of muscle disorder plus discopathy plus arthropathy only appeared in 7.8% of cases. It also bears mentioning that the mean values he obtained for discopathies and arthropathies not associated with any other pathology were 14% and 17.3%, quite higher than our values.

Manfredini (25) also observed the natural course of this pathology in patients reporting low pain intensity, who received advice on their signs and symptoms during their first visit, as well as suggestions on how to self-manage their symptoms; they were later assessed by the same person who diagnosed them, between 2 or 3 years later. He observed how the percentage of patients with muscle disorders decreased by 45%, disc displacements with reduction remained unchanged (52.1%), while 5.7% of patients suffering from disc displacement without reduction with limited opening showed no limitation. Arthralgias decreased by 16%, while osteoarthritis/osteoarthrosis remained almost unchanged. He concluded that the degree of correlation among the different groups and the gender variable was significant for a *p*-value ≥ 0.05 in the groups of muscle disorders and arthropathologies, which was not the case in the discopathy subgroup.

The higher values for the subgroups of muscle disorders and arthralgia in our study also coincides with the results recently reported by Kraus (26), obtained from a sample of 511 patients with OP and TMD who were referred to physical therapy. The highest value (79.6%) corresponded to the group of muscle disorders (62.2% without limited opening and 20% with limited opening), followed in frequency by arthralgia, with 46%, and 4.7% corresponding both to osteoarthritis and osteoarthrosis. Finally, there is discopathy, with quite lower values: 17%, 13% and 8% for DDWR, DDWOR with limited opening and DDWOR without limited opening, respectively. For this author, the most prevalent multiple diagnoses in Axis I in his sample are the group of muscle disorders in general, along with arthralgias.

As regards the differences between women and men in the distribution of all Axis I subgroups combined in our analysis, it bears stressing the statistically significant association between being a woman and suffering from a muscle disorder and/or joint disorder, but not from discopathies. This becomes more relevant if we take into account that both pathology subtypes are the only ones causing pain, while disc displacements do not always entail painful symptoms. This is in line with most studies in the literature, which do show a greater intensity of painful TMD symptoms in women than in men (15).

Among the main limitations of our study we must mention the lack of a control group, which is due to the fact that the sample was only obtained through patients who suffered from these disorders, due to the economic limitations and the time constraints for each visit, as the patients were examined in a public unit for diagnosing and treating OP and TMD that also provides other dental services. The possible bias in interpretation was avoided by having different researchers from those who obtained the sample data analyze them.

## Conclusions

In view of the results of our study and what has been found in the literature, we may conclude that the clinical subtypes most often found in patients with TMD signs and symptoms are those linked to muscle disorders, while joint and disc pathologies usually show a much lower proportion.

Regarding their different association with gender, the results confirm that there is a strong connection between being a woman and the presence of temporomandibular disorders, especially those clinical subtypes that are usually accompanied by painful symptoms, like muscle and/or joint pathologies.
